# Quality of life in dementia: a study on proxy bias

**DOI:** 10.1186/1471-2288-13-110

**Published:** 2013-09-06

**Authors:** Alexander MM Arons, Paul FM Krabbe, Carla JM Schölzel-Dorenbos, Gert Jan van der Wilt, Marcel GM Olde Rikkert

**Affiliations:** 1 Department for Health Evidence, Radboud University Medical Centre, PO Box 9101, 6500 HB, Nijmegen, The Netherlands; 2 Department of Epidemiology, University of Groningen, University Medical Center Groningen, PO Box 30.001, 9700 RB, Groningen, The Netherlands; 3 Multidisciplinary Memory Clinic Slingeland Hospital, Kruisbergseweg 25, 7009 BL, Doetinchem, The Netherlands; 4 Radboud Alzheimer Centre, Radboud University Medical Centre, PO Box 9101, 6500 HB, Nijmegen, The Netherlands; 5 Department of Geriatrics, Radboud University Medical Centre, PO Box 9101, 6500 HB, Nijmegen, The Netherlands

## Abstract

**Background:**

Measurement of health-related quality of life (HRQoL) in dementia is difficult. At some point people with dementia become unable to meaningfully assess their own HRQoL. At such a point in time researchers need to rely on other types of information such as observation or assessments from informal caregivers (proxies). However, caregiver assessments may be biased by several mechanisms. The current study explores whether caregivers project part of their own HRQoL in their assessments of patient HRQoL.

**Methods:**

The participants in the current study were 175 pairs, consisting of community-dwelling persons with dementia and their caregivers. The EQ-5D, the EQ-VAS and the QoL-AD were administered to collect HRQoL measurements from patients and caregivers at baseline, 6 months and 12 months. Two linear mixed models were used to investigate factors that bias proxy ratings, one with the EQ-VAS as dependent variable, and one with the EQ-5D utility as dependent variable. The independent variables were caregiver age, caregiver sex and caregiver QoL-AD items.

**Results:**

The linear mixed model with EQ-VAS as dependent variable indicated that 3 caregiver characteristics, namely caregiver age, money (caregiver’s financial situation) and valuation of life as a whole were significant predictors of the patient-by-proxy VAS scores. The linear mixed model with utility value as the dependent variable showed that caregiver age and valuation of the ability to do things for fun were significant predictors of the patient-by-proxy EQ-5D utility values.

**Conclusions:**

The current study was a first step in identifying factors that bias patient-by-proxy HRQoL assessments. It was discovered that caregivers project part of their own HRQoL onto patients when assessing patient HRQoL. This implies that patient-by-proxy HRQoL values should be interpreted with caution and not be used as a direct substitute for patient self-assessment, even when patients are no longer able meaningfully assess themselves.

## Background

Dementia describes a class of neurologic illnesses that cause progressive decline in cognitive functioning. Areas that are affected most severely are memory, reasoning, communication skills and the ability to carry out daily activities. In addition, people with dementia frequently suffer from behavioural and noncognitive symptoms such as depression, wandering, aggression, agitation, sleep disturbances, shouting, repeated questioning and psychosis [1].

Dementia is posing a great threat for the future of current health care expenditures as future scenarios claim that dementia prevalence may have doubled or tripled by 2050 [2,3]. With an increase in the number of patients with dementia, many governments will have to change their policies and focus on keeping patients out of nursing homes as long as possible. This is only possible with adequate pharmacological and psycho-social interventions. The evaluations of such programs should incorporate outcome measures such as health-related quality of life (HRQoL). Ideally, these HRQoL measures should be valid, reliable, and precise.

However, measurement of HRQoL in dementia is not without difficulties. First and foremost, the concept lacks a generally accepted definition. Several reviews have been published that describe the number and applicability of HRQoL instruments in dementia [4-7]. They show that more than a dozen dementia-specific instruments are available, each covering different domains and applying different methods of measurement.

Many researchers and clinicians argue that HRQoL is subjective in nature and thus only patient ratings should be considered valid. However, the very problem in dementia is that patients’ cognitive functioning decreases and therefore their ratings might become less valid or even unusable. Therefore, reliable and valid informal caregiver (proxy) ratings could be extremely useful in the field of dementia. Yet, using proxy ratings might have disadvantages.

Numerous studies report on agreement and differences between the dementia patients’ HRQoL ratings and those of patient-by-proxy [8-11]. They show a systematic underreporting of patient HRQoL by caregivers, as compared to patient self-assessment. These findings are in line with proxy reporting from other disease areas [12-19]. These studies identified factors that improved agreement between patients and caregivers. Such factors were higher patient education [20], a family caregiver (vs. other), lower levels of patient functional disability, higher levels of patient depression and lower caregiver burden [19], the type of perspective employed and higher patient cognitive functioning [13].

Dementia is a disease that not only affects patients but also their caregivers. Surprisingly, not much research has been done to identify caregiver characteristics that could influence agreement between patients and caregivers. Other than the type of caregiver, the perspective used [13], and caregiver burden [21], no caregiver characteristics have been identified. It has been acknowledged that providing care often results in personal, psychological, social and financial losses [22-26], although there are also care giving uplifts such as feelings of satisfaction, personal growth, enhancement and enrichment [27,28]. One could imagine that changes in these domains might have an influence on patient-by-proxy HRQoL ratings.

One study showed that caregiver HRQoL is related to the type of dementia and the coping style of the caregiver [29]. In addition, providing care for a patient with dementia is associated with a decline in HRQoL in both mental and physical domains [30]. Furthermore, it is unclear whether caregivers ‘project’ part of their own HRQoL problems onto patient HRQoL. For example, caregivers might underestimate the HRQoL of patients if they experience a diminished HRQoL themselves. This mechanism of caregiver ‘projection’ has been investigated in stroke [31] and other contexts such as end-of-life decisions [32,33]. In the latter context it is acknowledged that caregivers are imperfect decision makers and projection of caregivers’ preferences guide their decisions. If such a mechanism is present in patient-by-proxy HRQoL ratings then this is a type of bias of which clinicians and policy makers should be aware. The aim of this explorative study is to assess whether certain caregiver characteristics contribute to a bias of patient-by-proxy HRQoL assessments.

## Methods

### Respondents

The participants in the current study were dyads, consisting of community-dwelling persons with dementia and their informal caregivers, drawn from the AD-Euro RCT (a cost-effectiveness study on regular post-diagnosis care in dementia by memory clinics versus by general practitioners) [34,35]. The current study includes patients with a newly diagnosed dementia fulfilling DSM-IV-TR criteria and a score of 0.5-2 on the Clinical Dementia Rating (CDR; 0–3) scale: 0 for none; 0.5 for questionable/very mild; 1 for mild; 2 for moderate; and 3 for severe dementia [1,36]. In addition to the CDR, the Mini-Mental State Examination (MMSE) was administered, although scores on this instrument were not an inclusion or exclusion criterion [37]. Verbal and written consent of both the patient and caregiver were obtained when they were considered eligible at a screening in a multidisciplinary memory clinic. Patients were excluded if data collection was impossible (for example because of a severe hearing or language impairment), if they had a short life expectancy, if they were awaiting nursing home admission, or in case of a definite indication for specific memory clinic follow-up (for example having been diagnosed with a rare dementia). Data was collected by trained interviewers who administered the questionnaires and the response tasks at the patients’ homes. The instrument protocols were followed providing a standardized interview format across respondents. To maximize privacy and reduce potential bias, the person with dementia and the caregiver were interviewed separately. Interviews were conducted at a mutually convenient time. Measurements were obtained at baseline (T = 0), 6 months (T = 6) and 12 months (T = 12).

### Conceptual framework

Three perspectives to assess HRQoL were used, two of those were partially based on the work of Pickard and Knight [38]. First of all there is the patient self assessment (patient-patient perspective) of HRQoL. Second there is the assessment by the proxy of the patient’s HRQoL (patient-by-proxy perspective). In the current study caregivers were asked to rate patients from their own perspective. Pickard and Knight discuss one more perspective where proxies are asked to assess the patient as they think the patient would respond. This perspective was not used in the current study. Additionally, the caregiver’s own HRQoL was assessed (caregiver self-assessment).

### (Health-related) quality of life measures

#### QoL-AD

The QoL-AD is a dementia-specific quality of life (QOL) instrument [39,40]. It has 13 items covering the domains of physical health, energy, mood, living situation, memory, family, marriage, friends, self as a whole, chores, fun, money (financial situation), and life as a whole. Items are scored on a 4 point rating-scale ranging from 1 (poor) to 4 (excellent). There are separate versions available for patients and caregivers, and there is a version available for the caregiver’s own QOL [41]. The questions asked were: “How would you value your own (specific domains are mentioned here)?” for patient and caregiver self-assessments and “How would you value the person you care for his/her (specific domains)?” for patient-by-proxy assessments.

#### EQ-5D

The EQ-5D is a generic HRQoL instrument in which respondents evaluate their health state “today”. The classification system consists of five domains: mobility, self-care, usual activities, pain/discomfort and anxiety/depression, with three levels of severity per domain [42]. These levels indicate “no problems”, “some problems” or “severe problems”. The EQ-5D is the one of the most widely used index instruments, which is why it was chosen for this study. To calculate the values for the EQ-5D the well known UK tariff was used as described by Dolan [43]. The instrument also includes a vertical visual analogue scale (VAS) with a range of 0 (worst imaginable health state) to 100 (best imaginable health state). The EQ-5D was used for patient self-assessment, patient-by-proxy assessment and caregiver self-assessment.

### Analyses

Descriptive statistics of complete dyad data at T = 0, T = 6 and T = 12 were used to analyze patient-patient, patient-by-proxy and caregiver self-assessed HRQoL values. To compare outcomes on a 0–100 scale, EQ-5D utility scores were multiplied by 100 and QoL-AD sum scores (range 13–52) were transformed by applying the following formula:

$$\text{Scor}e_{\text{transformed}}=\left(\text{sum}\;\text{score}‒13\right)*2.564.$$

Intra-class correlation coefficients (ICC) were calculated to assess the level of substitutability between patient-patient and patient-by-proxy VAS and utility values. The strength of agreement between patients and caregivers is expressed as slight (ICC = 0.00-0.20), fair (ICC = 0.21-0.40), moderate (ICC = 0.41-0.60), substantial (ICC = 0.61-0.80) and almost perfect (ICC = 0.81-1.00).

The relationship of patient-by-proxy and caregiver self-assessed HRQoL values was investigated by looking at Pearson product–moment correlations coefficients (*r*) between their EQ-VAS scores. The EQ-VAS was selected for this analysis because this measure allows for the most subjective assessments, improving the ability to detect any biases. A significant correlation between the EQ-VAS scores would be interpreted as a potential projection bias in proxy assessments on patients. A similar analysis was performed on the EQ-5D utility values to investigate whether projection is also present in somewhat more objective assessments.

To investigate potential predictors of projection bias of proxy HRQoL onto patient HRQoL, a linear mixed model was used. Patient-by-proxy EQ-VAS was entered as a dependent variable, while for predictor variables caregiver age, sex, and caregiver self-assessed QoL-AD items were entered. These variables were used in the model at T = 0, T = 6 and T = 12. Age and sex were included in the model because these are very common confounders. In addition, the QoL-AD items were included to investigate which caregiver characteristics could bias patient-by-proxy assessments. The analysis was restricted to only these predictors because sufficient power to detect significant predictors needed to be preserved [44]. Here too a similar analysis was performed on the EQ-5D utility values. The dependent variable was the patient-by-proxy EQ-5D utility value, while caregiver age, sex and the caregiver self-assessed QoL-AD items were independent variables.

## Results

### Respondents

In total, 175 patients were included in the study, their descriptive statistics are provided below (Table 1). The sample consisted mostly of older patients with mild to moderate dementia. Most patients had a family caregiver, either a spouse or a child.

**Table 1:** Descriptive statistics of patient and caregiver dyads at T = 0, T = 6 and T = 12 months

		**T = 0 (n = 175)**		**T = 6 (n = 151)**		**T = 12 (n = 144)**	
		**n (%)**	**Mean (SD)**	**n (%)**	**Mean (SD)**	**n (%)**	**Mean (SD)**
**Patient**							
Age			78.6 (5.7)		78.5 (5.8)		78.4 (5.7)
Female		106 (60.6%)		90 (59.6%)		88 (38.9%)	
Type of dementia							
	Alzheimer	105 (60%)		93 (61.6%)		89 (61.8%)	
	Vascular	15 (8.6%)		9 (6.0%)		7 (4.9%)	
	Mixed	49 (28.0%)		43 (28.5%)		43 (29.9%)	
	Other	6 (3.4%)		6 (4.0%)		5 (3.5%)	
CDR							
	0.5	8 (4.6%)		8 (5.3%)		6 (4.2%)	
	1	139 (19.4%)		120 (79.5%)		117 (81.3%)	
	2	28 (16.0%)		23 (15.2%)		21 (14.6%)	
**Caregiver**							
Age			64.0 (13.2)		64.1 (13.2)		64.3 (13.2)
Female		123 (70.3%)		106 (70.2%)		101 (70.1%)	
Relation to patient							
	Spouse	94 (53.7%)		83 (55%)		80 (55.6%)	
	Child	72 (41.1%)		59 (39.1%)		55 (38.2%)	
	Other	9 (5.1%)		9 (6.0%)		9 (6.3%)	

### HRQoL outcomes

**Figure 1:**
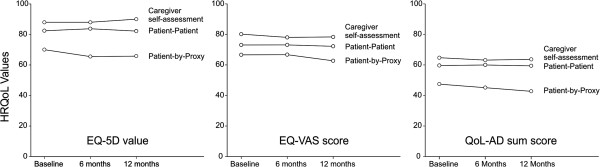
**EQ-5D values, EQ-VAS scores and QoL-AD sum scores (patient, caregiver, and patient-by-proxy) at baseline, 6 months and 12 months.** EQ-5D and QoL-AD values were rescaled between 0–100.

Caregiver self-assessed HRQoL assessments were the highest, followed by patient-patient and patient-by-proxy HRQoL assessments (Figure 1) on all three instruments at each time of measurement. EQ-5D values were the highest ($\bar{x}$ caregiver self-assessed = 88.59, $\bar{x}$ patient-patient = 82.77, $\bar{x}$ patient-by-proxy = 67.12), followed by VAS values ($\bar{x}$ caregiver self-assessed = 78.84, $\bar{x}$ patient-patient = 72.78 $\bar{x}$ patient-by-proxy = 65.33), followed by the QoL-AD values ($\bar{x}$ caregiver self-assessed = 63.86, $\bar{x}$ patient-patient = 59.65 $\bar{x}$ patient-by-proxy = 45.05).

### Patient-proxy substitutability

The ICC of patient-patient and patient-by-proxy VAS values were 0.18 at T = 0, 0.22 at T = 6 and 0.42 at T = 12. The ICC of patient-patient and patient-by-proxy utility values were 0.50 at T = 0, 0.46 at T = 6 and 0.51 at T = 12.

### Projection bias

There were significant correlations between the patient-by-proxy and caregiver self-assessed VAS scores on each of the three times of measurement. The correlations were 0.25 at T = 0 (*p* = 0.001), 0.43 at T = 6 (*p* < 0.001) and 0.20 at T = 12 (*p* = 0.012), thus indicating weak to moderate correlations. The correlations between the patient-by-proxy and caregiver self-assessed utility values were non-significant at each time of measurement (T = 0: *r* = 0.11, *p* > 0.1, T = 6: *r* = 0.13 *p* > 0.1, and T = 12: *r =* 0.12, *p* > 0.1).

The linear mixed model indicated that 3 caregiver characteristics, namely caregiver age, money (caregiver’s financial situation) and valuation of life as a whole were significant predictors of the patient-by-proxy VAS scores (Table 2). This model had a combined 498 observations across the three measurement times. A total of 407 observations were used in the model. The linear mixed model with utility value as the dependent variable showed that caregiver age and valuation of the ability to do things for fun were significant predictors of the patient-by-proxy EQ-5D utility scores (Table 3). This model had a combined 525 observations across the three measurement times. A total of 486 observations were used in the model.

**Table 2:** Proxy characteristics as predictors of patient-by-proxy VAS scores in a linear mixed model

	**β-Coefficient**	**p-value**	**95% confidence interval**
Intercept	51.26	0.000	25.48 - 77.05
Proxy sex	0.45	0.852	−4.34 - 5.25
**Proxy age**	**0.19**	**0.033**	**0.02 - 0.36**
Physical health	−0.32	0.846	−3.59 - 2.94
Energy	1.70	0.294	−1.49 - 4.90
Mood	0.22	0.901	−3.30 - 3.74
Living situation	−0.82	0.611	−3.97 - 2.34
Memory	−2.28	0.271	−6.35 - 1.79
Family	−1.04	0.524	−4.24 - 2.17
Marriage	1.09	0.498	−2.08 - 4.26
Friends	1.15	0.493	−2.14 - 4.44
Self as a whole	−0.96	0.645	−5.08 - 3.16
Ability to do chores around the house	0.96	0.520	−1.98 - 3.91
Ability to do things for fun	0.84	0.539	−1.85 - 3.52
**Money**	**−5.53**	**0.005**	**−9.37 - −1.70**
**Life as a whole**	**5.78**	**0.004**	**1.84 - 9.71**

R^2^ = 0.068 (R^2^ compared to an intercept only model).

**Table 3:** Proxy characteristics as predictors of patient-by-proxy EQ-5D utility values in a linear mixed model

	**β-Coefficient**	**p-value**	**95% confidence interval**
Intercept	0.07	0.734	−0.04 - 0.53
Proxy sex	−0.01	0.760	−0.10 - 0.07
**Proxy age**	**0.01**	**0.000**	**0.00 - 0.01**
Physical health	0.04	0.154	−0.02 - 0.10
Energy	0.04	0.159	0.02 - 0.10
Mood	−0.05	0.128	−0.11 - 0.01
Living situation	0.02	0.507	−0.04 - 0.07
Memory	−0.04	0.304	−0.11 - 0.03
Family	0.01	0.834	−0.05 - 0.06
Marriage	0.01	0.632	−0.04 - 0.07
Friends	0.00	0.926	−0.06 - 0.07
Self as a whole	0.01	0.733	−0.06 - 0.08
Ability to do chores around the house	−0.02	0.526	−0.07 - 0.04
**Ability to do things for fun**	**0.05**	**0.041**	**0.00 - 0.10**
Money	−0.03	0.417	−0.09 - 0.04
Life as a whole	0.03	0.417	−0.04 - 0.01

R^2^ = 0.095 (R^2^ compared to an intercept only model).

## Discussion

The current study attempted to explore the existence of potentially biasing factors that might influence patient-by-proxy HRQoL assessments in dementia. A significant correlation between patient-by-proxy and caregiver self-assessed EQ-VAS scores was found. This correlation remained statistically significant over time. Moreover, characteristics of caregivers were identified that bias their VAS ratings on patients.

A biasing factor on VAS ratings that was identified was ‘life as a whole’. This factor has at face value a great contribution to projection. Caregivers incorporate part of their overall assessments of their own lives into the assessments of the patients’ lives. Should this finding be replicated then such a bias can be overcome by measuring caregiver HRQoL alongside patients. Researchers might then adjust the ratings of caregivers on patients by using a correction algorithm.

Another biasing factor on VAS ratings that was identified was ‘money’. This factor contributes strongly to the overall rating. As this estimate was negative this implies that the better the caregivers’ financial situation are, the worse their ratings on patient HRQoL will be and vice versa. This is a new finding and to the authors’ knowledge has not been previously reported elsewhere. These results seem counterintuitive, as previous research has demonstrated that having more or enough money would improve (HR)QoL [45]. A possible explanation for this relationship could be that financial status functions as a mediating variable for socio-economic status (SES). Caregivers with a higher SES might perceive the impact dementia has on the patient and themselves to be bigger than lower SES proxies. This might indicate that caregivers with higher SES perceive more shame and experience the difference in HRQoL between themselves and the patient to be larger. More research is needed to further explore this finding.

The least strong characteristic was caregiver age. Age contributed little to the overall rating, but older caregivers gave higher ratings. This finding is in line with general findings that aspects of QOL such as happiness are rated higher as age increases [46,47]. Moreover, because spousal caregivers are generally older than child caregivers, it is highly likely that spousal caregivers rate patients higher than child caregivers do. If such is the case, then this might have serious implications for outcomes research. For example, if a new study is to be initiated to evaluate a new intervention which uses spousal caregivers to assess the HRQoL of people with dementia, such a study might overestimate the effects on HRQoL when compared to a study that solely uses child caregivers. Further research is required to corroborate this finding. It is acknowledged that age is one of the most common confounders in observational research. Nevertheless, clinicians and policy makers should be made aware of its potentially biasing effects on HRQoL ratings.

There were two biasing factors on utility values, caregiver age and ‘ability to do things for fun’. The implications for caregiver age are similar to the VAS ratings that were discussed previously. Interestingly, the ability of caregivers to do things for fun biases their assessments of the EQ-5D items on patients. Since the β-coefficient is positive this means that caregivers who are better able to do things for fun give better ratings of patient functioning. One possible explanation for this phenomenon might be that caregivers who undertake many fun activities with a patient also think that the patient experiences a similar level of fun. It might thus be possible that the amount of fun caregivers experience bias their assessment of patient functioning.

These newly identified factors differ from those previously identified in other research areas. For example, patient depression has previously been identified as a factor leading to an increase in patient and proxy differences for elderly patients visiting the emergency room [38]. In addition, burden and psychological distress in caregivers was a significant predictor of patient and proxy differences in psychosocial scores in veterans [48]. A different study [49] that focused more on functional status through (instrumental) activities of daily living identified the following factors that contribute to more disagreement between patients and proxies: female proxies, proxies who lived with the patient, proxies who were not first-order relatives of the patient, and proxies who assisted patients with (instrumental) activities of daily living. The newly identified factors thus provide fruitful grounds for new research on systematic differences between patient and proxy assessments.

It should be noted that the explained variance of both linear mixed models was low. However, with the current study design, this is a desirable outcome. If the models would explain all of the variance then this would imply that patient-by-proxy assessments would only be based on proxy characteristics and not on patient characteristics. In this study the explained variance in the models was less than 10% which suggests that the bias that is present in patient-by-proxy HRQoL assessments is small compared to the influence of actual patient characteristics.

In general, measurement of HRQoL in dementia is difficult. In other disease areas patient self-assessment is usually regarded as a gold standard against which proxy assessment is compared. HRQoL is a very subjective concept and thus patients have ‘privileged access’. In dementia however, researchers have questioned the assumption that people with dementia should be regarded as the gold standard since cognitive impairments might lead to less valid self-assessments. For example, Lawton [50] noted “most cognitively impaired patients do not introspect, or at least do not report reliably on interior phenomena”. However, the authors feel that patients’ self-assessment is the best measure of HRQoL, as long as the patients can deliver this measure [51]. If patients cannot give their HRQoL assessment anymore, one has to rely on proxy measures. However, since cognitive functions are primarily affected by dementia, caregivers may be less capable of assessing the internal state of the dementia patients they care for and therefore they might provide less valid HRQoL assessments compared to other disease areas.

Patient-by-proxy assessments can be used for two distinct purposes. The first is substitution of patient self-assessment. In this situation the patient self-assessment is considered a gold standard to compare patient-by-proxy assessment with. However, when patient-by-proxy assessments are used in addition to patient self-assessment, for example to provide extra information for clinical decision making, then patient-self-assessment should not be considered a gold standard. In this context, proxy reporting might even be more valid than patient-self assessment as the disease progresses. Nevertheless, the biases that were identified might occur in both substitution judgments and informing clinical decision making.

One major limitation of the current study is that the proxy perspective investigating how HRQoL is *according to the patient* was not measured. We therefore cannot conclude whether or not caregivers actually know how patients would assess themselves. The addition of this perspective could provide additional information on caregiver bias, since it might be different when multiple perspectives are used [52]. In addition, the relatively short follow up time and the relatively homogenous sample make it difficult to generalize the current findings to a broader context.

## Conclusion

Proxy assessment is an important aspect of the evaluation of people with dementia, yet a bias is present. Many caregivers might report on different aspects than patients, and thus the patient self-assessment and patient-by-proxy assessment perspectives might be complementary instead of being regarded interchangeable. However, this makes it more difficult to deal with the proxy bias that is present as shown in this study. Proxy bias might have serious implications for clinical and policy decisions. Dementia is a progressive disease, in which patients at some point become unable to express their HRQoL in a meaningful and valid way. Therefore, after such a point in time, one has to consider alternatives such as proxies or behavioural observations. Nonetheless, if the bias found in this study is generalizable to broader contexts, clinicians and policy makers should be made aware of its influence on proxy HRQoL assessments. For future studies we recommend measurement of HRQoL of patients and proxies, with identical instruments, and multiple perspectives. Should future studies discover more complete causal models of reported HRQoL values, it might then be possible to constrain proxy biases to a minimum.

### Ethical approval

The study was approved by the Medical Ethics Committee of the Radboud University Medical Centre.

## Competing interests

Marcel Olde Rikkert is consultant for Numico, ECHO Pharmaceuticals, and was consultant for Novartis, Janssen-Cilag, and Schering-Plough. Marcel Olde Rikkert’s fees for presentations and honoraria were only paid to the institution. Marcel Olde Rikkert has no financial interests that conflict with the integrity for this paper. All other co-authors had no competing interests, financial or otherwise.

## Authors’ contributions

MOR and PK made substantial contributions to the AD-Euro study design which the current manuscript draws its data from. CSD made substantial contributions to data collection. AA was responsible for the analyses and interpretation and drafting the initial manuscript. MOR, PK, CSD, GJvdW, and AA subsequently contributed to critically revising the manuscript. All authors read and approved the final manuscript.

## Pre-publication history

The pre-publication history for this paper can be accessed here:

http://www.biomedcentral.com/1471-2288/13/110/prepub

## Data sharing

The raw dataset is available from the corresponding author at s.arons@ebh.umcn.nl. Participants’ consent was not obtained, but the data presented are anonymised and risk of identification is low.
